# Low-G Triggered Acceleration Switch for Near-Zero Power Wake-Up Application

**DOI:** 10.3390/mi13081333

**Published:** 2022-08-17

**Authors:** Yingzhou Han, Guozhe Xuan, Jiahao Zhao, Zheng You

**Affiliations:** 1 State Key Laboratory of Precision Measurement Technology and Instruments, Beijing 100084, China; 2 Beijing Advanced Innovation Center for Integrated Circuits, Beijing 100084, China; 3 Department of Precision Instrument, Tsinghua University, Beijing 100084, China

**Keywords:** MEMS, acceleration switch, wake up, low-g, near-zero power

## Abstract

A low-g triggered micro-electromechanical system (MEMS) resonant acceleration switch is designed, fabricated and tested in this paper for near-zero power wake-up applications. The switch is actuated by ambient low-g vibration, consuming zero power while waiting for vibration at its resonant frequency. A cantilever beam and proof mass structure is adopted in the switch. The patterns of spiral cantilever beams are designed for low resonant frequency and threshold. Once the vibration with resonant frequency exceeds the acceleration threshold of the switch, the movable electrode becomes sufficiently displaced to contact the fixed electrodes and causes them to trigger. The dynamic responses of the switch are tested on a piezoelectric stack. The experimental results show that the switch closes under vibration at a frequency as low as 39.3 Hz and at an acceleration threshold of 0.074 g. A wake-up sensor node connected to the switch can awaken when the switch is under vibration as an intended characteristics.

## 1. Introduction

Wireless sensor networks (WSNs) are now widely used in various areas such as in environmental monitoring and intrusion detection [1]. However, further applications of WSN are limited by their power. Sensor nodes powered by batteries can only work continuously for several months, and it is inconvenient to replace batteries for large numbers of sensor nodes [2,3].

Reducing ineffective operating time is a possible way to save power when sensor nodes are used to monitor the environment, so that the lifespan of these sensor nodes is extended [2]. State-of-the-art sensor nodes are provided with isolated sensing modules to monitor concerned events, such as sound [4,5,6], infrared radiation [7,8], temperature [9] and vibration [10,11,12,13]. They awaken only when the event occurs. Such an approach can ensure that the systems are kept in sleep mode most of the time, in order to increase their lifespan.

Vibration is a useful target among all these signals as it reflects the activities of vehicles and people located nearby [14]. MEMS accelerometers have been used in previous studies in order to monitor ambient vibration. However, these kinds of sensors must be powered continuously [14,15,16]. Recent works used near-zero power sensors in order to overcome this disadvantage. Piezoelectric material was used in some near-zero power sensors [10,11,12]. The piezoelectric sensors resonate at a specific frequency vibration, transforming kinetic energy into electrical signals for wake-up applications. An aluminum nitride (AlN) piezoelectric MEMS accelerometer is presented to monitor ground vibration [10]. It is designed to resonate at the target frequency (160 Hz), and a voltage sensitivity for acceleration of 26 V/g is then achieved. However, the accelerometer requires a complicated Complementary Metal Oxide Semiconductor (CMOS) circuit in order to convert the sine voltage into a step signal. Another approach is by using an acceleration switch that closes when the intended vibration is detected. Most acceleration switches respond to an acceleration over 2 g [17,18,19], so they are not practical in environmental monitoring as the ambient vibration is usually less than 0.1 g. A rotational design MEMS resonant acceleration switch is explored to respond to vibration at frequencies between 30 Hz and 1000 Hz [13]. The switch closes at a vibration lower than 1 g with a resonant frequency and consumes less than 0.1 nW when no vibration is presented. However, the switch needs to be electrostatically tuned in order to reach a low resonant frequency that increases power consumption. Thus, a switch that can be triggered by a low-frequency and low-amplitude vibration and that does not require additional circuits or power in a standby state is needed for wake-up applications.

A resonant MEMS acceleration switch is described in this paper for wake-up applications. The switch is designed to close under vibration at a specific frequency so that it can identify targets. A cantilever beam and proof mass structure is installed in the switch, with beams designed in a spiral shape. A movable electrode is placed on the back of the proof mass while the fixed electrodes are set on a glass substrate. The proof mass moves in an out-of-plane direction generated by excitation. Different spiral shapes are compared in this paper and a more appropriate pattern is chosen for a lower resonant frequency and threshold in a specific area of the device. The switch is fabricated using silicon-on-glass bonded wafers. As the experimental results on a shaker system show, the switch can be triggered at a frequency as low as 39.3 Hz. The acceleration threshold at a resonant frequency is also as low as 0.074 g. The switch was placed inside a sensor node so that it consumes near-zero power in its standby state while the switch monitored the ambient vibration. The main advantage of the switch proposed in this paper is that it can be triggered by a low-g vibration at a specific frequency, thereby preventing false wake-ups, without any extra power consumption in its standby state. This novel research is meaningful for extending the operational life of the sensor nodes.

## 2. Design

**Figure 1:**
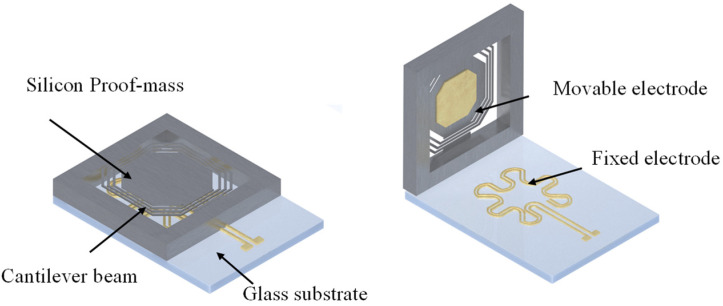
Diagrammatic structure of the resonant acceleration switch.

The switch is designed to close when stimulated by weak vibration with a resonant frequency of the switch. As shown in Figure 1, there are four parts to the switch: a cantilever beam, a proof mass, a glass substrate and electrodes. The spiral beams are used to lower the resonant frequency of the switch. A movable electrode is attached to the back side of the proof mass to contact the fixed electrodes on the substrate when it resonates after sufficient displacement.

**Figure 2:**
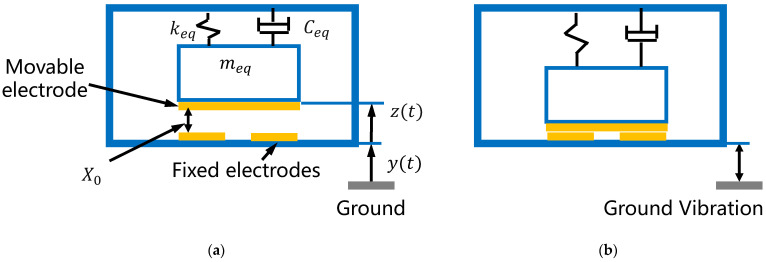
Cross section of the resonant acceleration switch. (**a**) Standby state; (**b**) closed state.

As shown in Figure 2a, the metal electrode on the back of the silicon proof mass is set apart from the fixed electrodes in the switch’s standby state. When the switch is stimulated by ground vibration at a specific frequency, as in Figure 2b, the proof mass moves downward by a sufficient distance for the movable electrode to contact the two fixed electrodes, so that the two fixed electrodes are connected.

The cantilever beam–proof mass structure in Figure 2a can be simplified as a spring-mass-damping system with this kinetic equation [20]:

(1) $$m_{eq}\ddot{z}+C_{eq}\dot{z}+k_{eq}z=-m_{eq}\ddot{y}$$

where z is the displacement of a point on the cantilever beam relative to the glass substrate, y is the absolute displacement of the substrate, $m_{eq}$ is the equivalent mass, $C_{eq}$ is the damping coefficient and $k_{eq}$ represents spring stiffness. The inertial force on the spring-mass-damping system is $-m_{eq}\ddot{y}$.

The resonant displacement of the proof mass under a simple harmonic excitation $y\left(t\right)=Y_{0}e^{j\omega t}$ is

(2) $$z\left(t\right)=\frac{\omega^{2}}{\omega_{res}^{2}-\omega^{2}+j2\zeta \omega_{res}\omega }Y_{0}e^{j\omega t}$$

where ω is the frequency of the excitation, $\omega_{res}$ is the resonant frequency, ζ is the mechanical damping coefficient and $Y_{0}$ is the magnitude of the displacement. So, the resonant frequency and the acceleration threshold $a_{th}$ can be represented by

(3) $$\omega_{res}=\sqrt{\frac{k_{eq}}{m_{eq}}}$$

(4) $$a_{th}=2Y_{0}X_{0}\zeta \omega_{res}^{2}$$

where $X_{0}$ is the initial distance between the movable electrode and the fixed electrodes. From Equations (3) and (4), the acceleration threshold of the switch is influenced by the resonant frequency, which is determined by the equivalent mass and stiffness. The threshold is also related to the initial distance between the electrodes.

**Figure 3:**
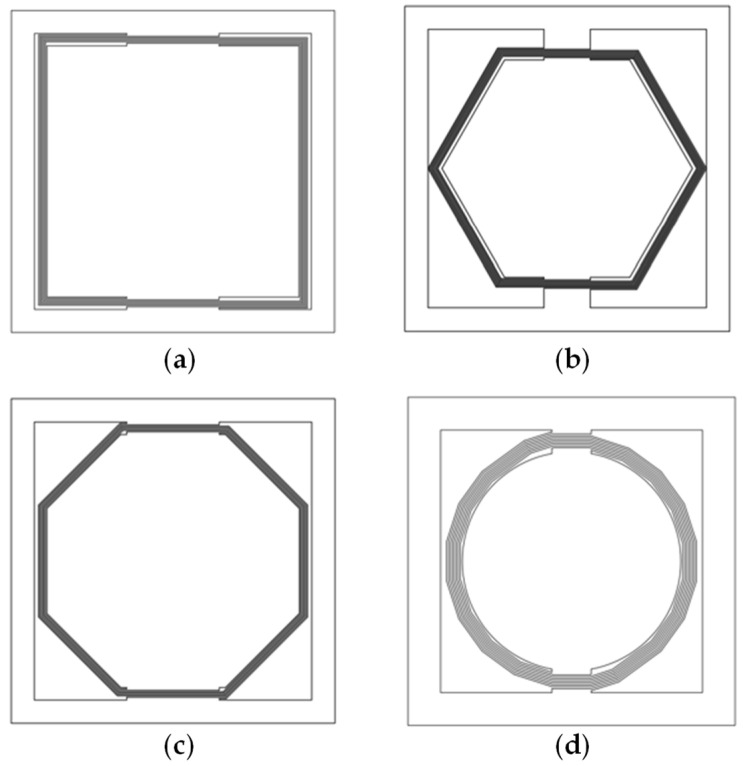
Top view of the different patterns of cantilever beams. (**a**) Square pattern; (**b**) regular hexagon pattern; (**c**) regular octagon pattern; (**d**) circle pattern.

To achieve a low resonant frequency and a low acceleration threshold, the patterns of the cantilever beams and the proof mass should be carefully evaluated. These are usually spiral beams. For the acceleration switches with spiral beams that are limited in a specific occupied area, several patterns of cantilever beams are shown in Figure 3. The key point of the switch design is to figure out which kind of pattern can achieve our goals in a limited space with an easy fabrication process.

**Figure 4:**
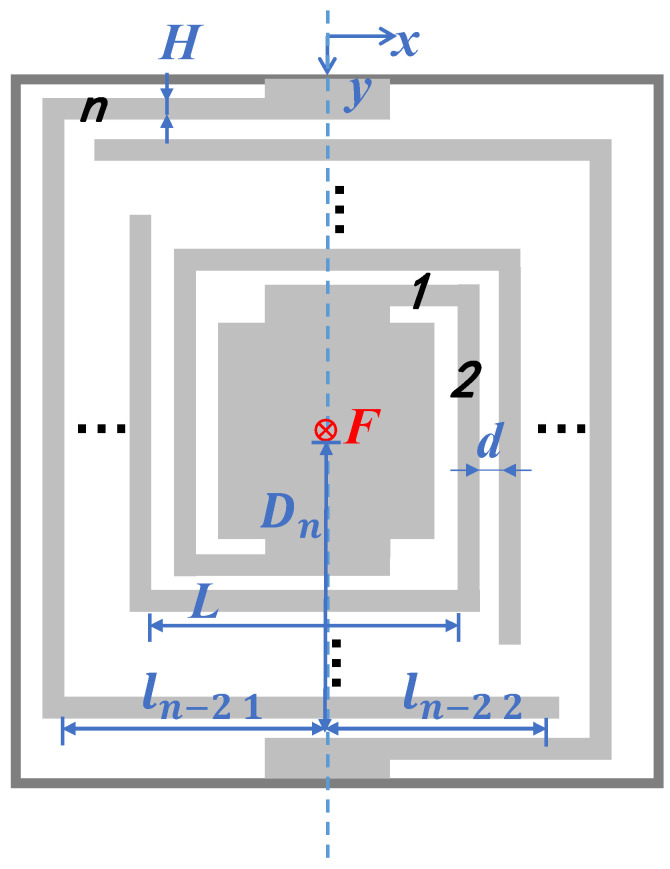
Diagram defining the dimensional variables of the square pattern.

The definitions of the beam parameters in the square pattern are shown in Figure 4 and Table 1. The beams are subjected to both torque and bending moments while vibrating, so the displacement of the proof mass δ under force F can be derived using Karl’s theorem.

(5) $$\delta =\frac{\partial U}{\partial F}=\int^{\;}\frac{T\left(x\right)}{GI_{p}}\frac{\partial T\left(x\right)}{\partial F}dx+\int^{\;}\frac{M\left(x\right)}{EI_{s}}\frac{\partial M\left(x\right)}{\partial F}dx\;$$

(6) $$I_{p}=WH^{3}$$

(7) $$I_{s}=\frac{WH^{3}}{12}$$

where U is the strain energy of the beams, and T and M are the torque and bending moments, respectively.

As L≫W+d, the beams in one turn can be considered as four straight beams with equal lengths. Each beam can be divided into $l_{i1}$ and $l_{i2}$ with the vertical line as the dividing point. So,

(8) $$D=\frac{A}{2}-\left(n+1-i\right)d-\frac{2\left(n-i\right)+1}{2}W$$

D is the vertical distance between the beam and the center of gravity of the proof mass.

As a result, the torque and bending moments along beam $l_{i1}$ can be presented as

(9) T(x)=FD(0≤x≤D)M(x)=Fx(0≤x≤D)

As there two centrosymmetric beams, the displacement $\delta_{i1}$ of beam $l_{i1}$ under force F is

(10) $$\begin{array}{ll} \delta_{i1} & =\frac{\partial U}{\partial F}=\int^{\;}\frac{T\left(x\right)}{GI_{p}}\frac{\partial T\left(x\right)}{\partial F}dx+\int^{\;}\frac{M\left(x\right)}{EI_{s}}\frac{\partial M\left(x\right)}{\partial F}dx\\ & =\int_{0}^{D}\frac{FD}{GI_{p}}\cdot D\cdot dx+\int_{0}^{D}\frac{Fx}{EI_{s}}\cdot x\cdot dx\;\\ & =\frac{1}{2}FD^{3}\left(\frac{1}{GI_{p}}+\frac{1}{3EI_{s}}\right)\;\;\; \end{array}$$

So, the total displacement of the proof mass is

(11) $$\delta_{4}=\overset{n}{\underset{i=1}{\sum }}8\delta_{i1}$$

The stiffness of beams $k_{eq}$ can be represented by the displacement of the proof mass δ under force F:

(12) $$k_{eq}=\frac{F}{\delta_{4}}$$

As $m_{beam}\ll m_{mass}$, thus

(13) $$m_{eq}\approx m_{mass}$$

Then, the resonant frequency of the switch is defined by its geometric parameters:

(14) $$f=\frac{1}{2\pi }\sqrt{\frac{k_{eq}}{m_{eq}}}=\frac{1}{2\pi }\sqrt{\frac{F}{m_{mass}\sum_{i=1}^{n}8\delta_{i1}}}$$

Other patterns of beams can be calculated with similar methods.

(15) $$\delta_{i1}=\frac{1}{2}F\left(L_{2}-L_{1}\right)\left[\frac{D^{2}}{GI_{p}}+\frac{L_{2}^{2}+L_{1}^{2}+L_{1}L_{2}}{3EI_{s}}\right]=\frac{1}{2}FL_{2}\left(\frac{D^{2}}{GI_{p}}+\frac{L_{2}^{2}}{3EI_{s}}\right)$$

(16) $$\delta_{6}=\overset{n}{\underset{i=1}{\sum }}12\delta_{i1}$$

(17) $$\delta_{i1}=\frac{1}{2}F\left(L_{2}-L_{1}\right)\left[\frac{D^{2}}{GI_{p}}+\frac{L_{2}^{2}+L_{1}^{2}+L_{1}L_{2}}{3EI_{s}}\right]=\frac{1}{2}FL_{2}\left(\frac{D^{2}}{GI_{p}}+\frac{L_{2}^{2}}{3EI_{s}}\right)$$

(18) $$\delta_{8}=\overset{n}{\underset{i=1}{\sum }}16\delta_{i1}$$

(19) $$\delta_{c}=\frac{1}{2}F\left(L_{2}-L_{1}\right)\left[\frac{D^{2}}{GI_{p}}+\frac{L_{2}^{2}+L_{1}^{2}+L_{1}L_{2}}{3EI_{s}}\right]=\pi DF\left[\frac{D^{2}}{GI_{p}}+\frac{{\left(2\pi D\right)}^{2}}{3EI_{s}}\right]$$

**Figure 5:**
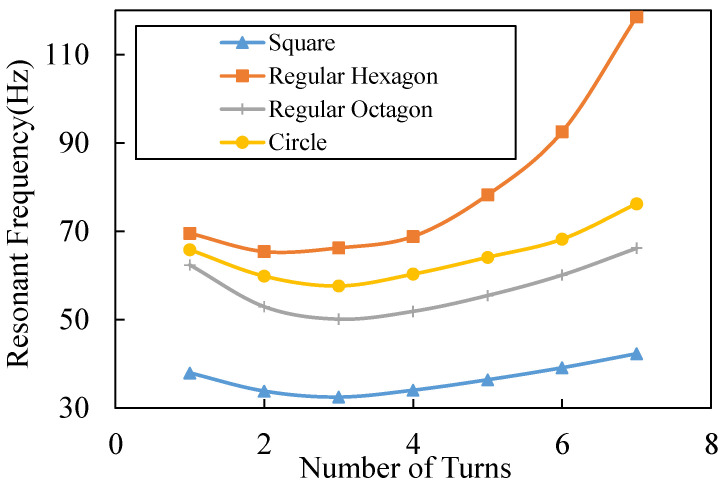
Analytical results of the resonant frequencies versus the number of turns of the beams in different patterns.

From Equations (10) and (14), we understand that reducing the width and thickness of the cantilever beams can lower the resonant frequency of the switch. However, this paper will not focus on such parameters as they are mainly determined by fabrication ability. Here, we mainly focus on the impact of different patterns. Proper patterns can increase the mass of the proof mass and the length of the cantilever beams, which also lead to a lower resonant frequency and a lower acceleration threshold, as Equation (14) shows. The resonant frequencies of the switches with different shapes of beams are calculated in MATLAB 2017 (Natick, MA, USA), as shown in Figure 5; the size of each switch is limited in 6 ∗ 6 mm; the thickness of beams and the proof-mass is set at 50 μm; and the width and intervals of the adjacent beams are set at 40 μm and 20 μm separately, which are out of fabrication consideration. The resonant frequency of the switch with the square spiral beams is the lowest, while the switch with the circle spiral beams is the highest. The reason for such results is because the square spiral pattern has a lower stiffness of beams and a larger mass because of the greater utilization of space. The switch with the three-turn square spiral beams achieves the lowest resonant frequency 31 Hz, which is only 56% of the lowest resonant frequency of the circle spiral pattern.

**Figure 6:**
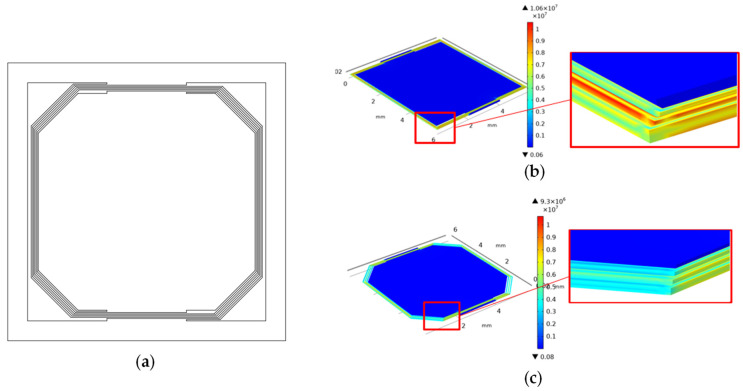
(**a**) Square spiral pattern with chamfers at the corners; (**b**) simulation results of the stress distribution of the square spiral pattern; (**c**) simulation results of the stress distribution of the square spiral pattern with the chamfers at the beam corners.

However, the disadvantage of the square spiral pattern is the stress concentration at its corners, which may cause the switch to break during fabrication or use. Chamfers are added at the corners of the beams in order to solve this problem, as shown in Figure 6a. COMSOL Multiphysics 5.4 (Stockholm, Sweden) simulation results of the stress distribution are shown in Figure 6b,c. Details of simulation are described in Appendix A. The maximum stress at the corners of the square spiral pattern is over 10.6 MPa, yet that of the circle spiral pattern is only 3.4 MPa. If chamfers are added at corners of the square spiral beams, the maximum stress at the corners will decrease to 6.6 MPa, as shown in Figure 6c.

**Figure 7:**
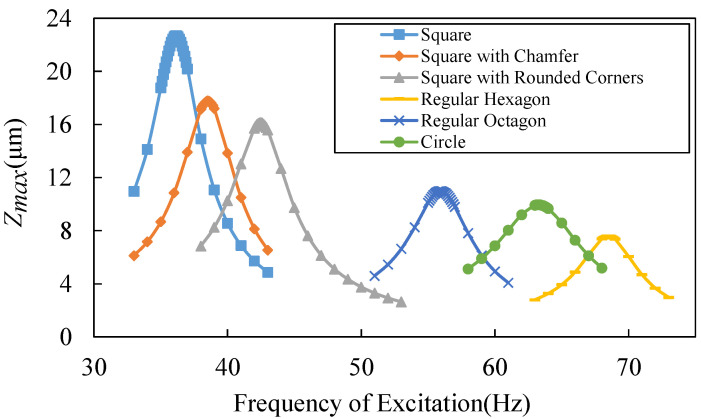
Simulated relative displacement versus different excitation frequency and beam patterns.

The resonant frequencies and maximum displacements of the proof mass relative to the basement are also simulated, as shown in Figure 7. The amplitude of excitation is 0.01 g and the damping ratio is 0.1 in the simulation model. The damping ratio is an approximation that is obtained by comparing measurement results for the proof mass displacement with the simulation results. The geometric parameters of the devices are shown in Table 2. All of the devices are designed as two spiral beam circles around the proof mass. The switch with chamfered beams has little to sacrifice in the resonant frequency and the maximum displacement compared to the square spiral pattern switch, as shown in Figure 7. The maximum stress at the corner is decreased by nearly 40%, as shown in Figure 6. As a result, the square spiral pattern with chamfered corners is a better design, as it reaches a balance between a low resonant frequency and a low stress concentration.

**Figure 8:**
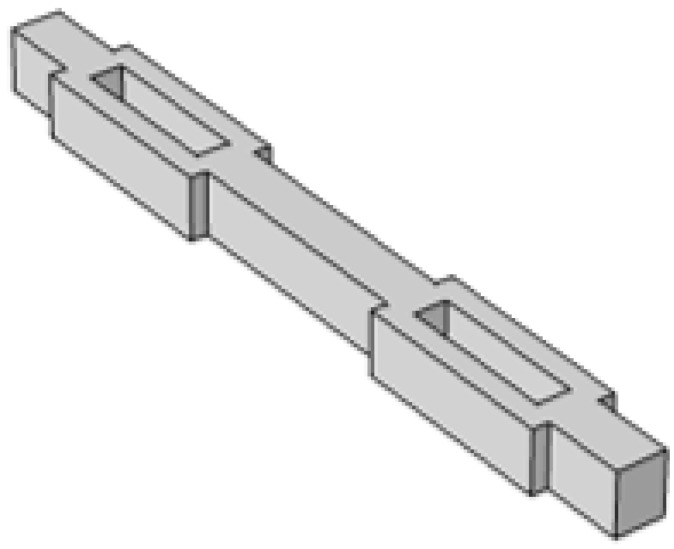
Design of hollow beams.

**Figure 9:**
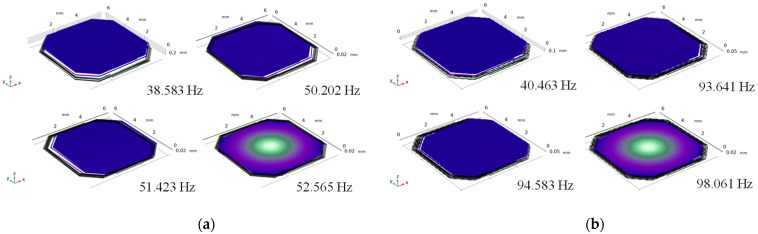
(**a**) Modal simulation results of normal beams; (**b**) modal simulation results of hollow beams.

Another fatal factor that prevents the MEMS structure from resonating at a low frequency is that the first-order eigenfrequency and the second-order eigenfrequency may become too close to influence the vibration direction of the silicon proof mass. The reason for this is because the limited volume of the MEMS device makes the out-of-plane stiffness and the in-plane stiffness of the silicon beams become close. The hollow beam is used here to separate the first-order eigenfrequency from the second-order as it enlarges the in-plane stiffness of the beams with little influence on the out-of-plane stiffness, as shown in Figure 8. The simulation results of the eigenfrequencies are shown in Figure 9. The first-order eigenfrequency of the normal beam switch is 38.6 Hz and the second-order eigenfrequency is 50.2 Hz, which are too close. Figure 9b shows the simulation results after the hollow beams are used in the device. The second-order eigenfrequency changes from 50.2 Hz to 93.6 Hz, while the first-order eigenfrequency stays at around 40 Hz.

## 3. Fabrication

**Figure 10:**
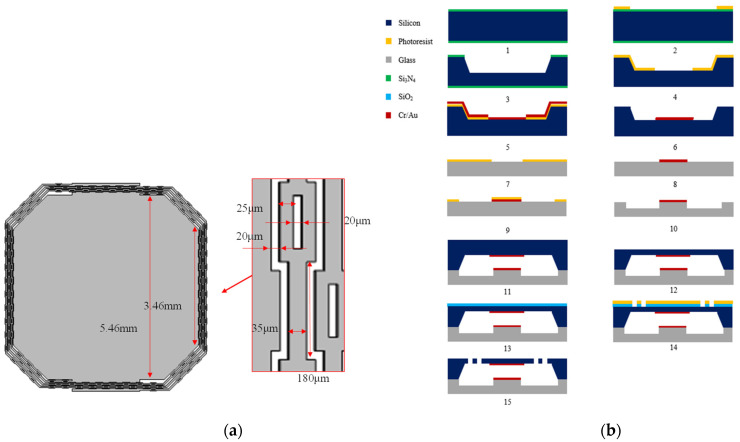
(**a**) Detailed size of the fabricated device; (**b**) Fabrication process of the device.

The overall device fabrication comprises fifteen steps, as shown in Figure 10. A silicon wafer with a thickness of 400 μm was used in the fabrication. After initial cleaning, the wafer was wet etched 200 μm to form a cavity for the proof mass moving. Chromium and gold were deposited and patterned on the cavity surface of the silicon wafer, with 50 nm and 350 nm thicknesses, respectively, by evaporation and the lift-off process. The size of the electrode is 3 mm × 3 mm. The 50 nm-thick chromium and 350 nm-thick gold were also deposited and patterned on a 500 μm-thick glass substrate to form fixed electrodes. The two parallel fixed electrodes were designed as a special shape for better contact, as shown in Figure 1. The glass substrate was wet etched 0.7 μm in the next step in order to prevent the silicon proof mass from adhering to the glass substrate after it was released. Then, the silicon wafer was bonded onto the glass substrate. This was followed by chemical polishing in order to thin the silicon wafer down to 250 μm thickness. Finally, an inductively coupled plasma (ICP) etching was used on the silicon wafer in order to release the device forming the graphics of the switch.

**Figure 11:**
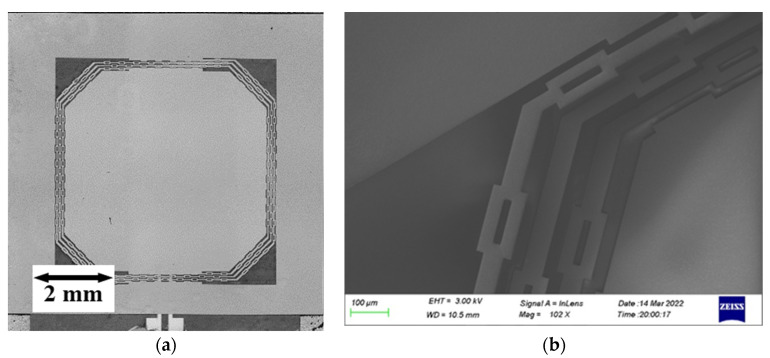
(**a**) Optical photograph of the resonant acceleration switch; (**b**) SEM of the beams of the resonant acceleration switch.

An optical photograph and SEM image of the switch are shown in Figure 11. The structure of the switch was released without damage or adhesion. The proof mass sags 163 μm under the gravity measured by a white light interferometer, which is 4.9% deviated from the simulated value 155 μm. The experimental result is basically the same as the simulated result.

## 4. Testing

**Figure 12:**
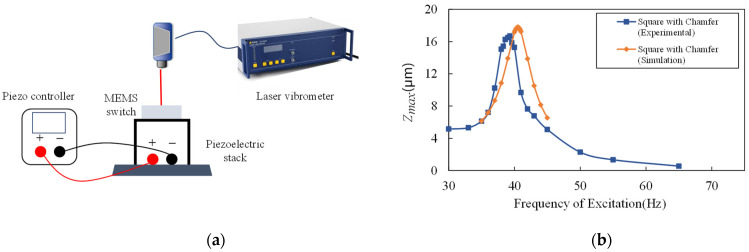
(**a**) The experimental setup of the piezoelectric stack; (**b**) comparison of the simulation result and the experimental result of the relative displacement versus the excitation frequency.

A piezoelectric stack (THORLABS, Newton, United States) and a Laser Doppler Vibrometer (LDV, Polytec, Karlsruhe, Germany) were used to measure the dynamic behavior of the switch. The LDV system measures the out-of-plane absolute displacement of the proof mass when the device is excited by a piezoelectric stack. The input voltage of the piezoelectric stack is recorded at the same time in order to calculate the absolute displacement of the substrate. The experimental set is shown in Figure 12a. The relative displacement Z can be calculated by the absolute displacement of the proof mass minus the absolute displacement of the substrate.

As shown in Figure 12b, the amplitude–frequency characteristics were measured in the air while excitation of the switch is 0.01 g at the different frequencies. The resonant frequency of the switch is 39.3 Hz, a 3% deviation from the simulation result 40.5 Hz. The maximum relative displacement between the proof mass and the substrate is 16.7 μm, a 6.6% deviation from the simulation result, which was 17.8 μm. The experimental result is basically consistent with the simulated value. The deviation is speculated to be caused by fabrication errors, which lead to narrowed cantilever beams. Compared with the simulation results, the experimental result also verifies that the resonant frequency of the switch under this pattern is lower than that of other patterns under the same processing level and the same occupied area.

**Figure 13:**
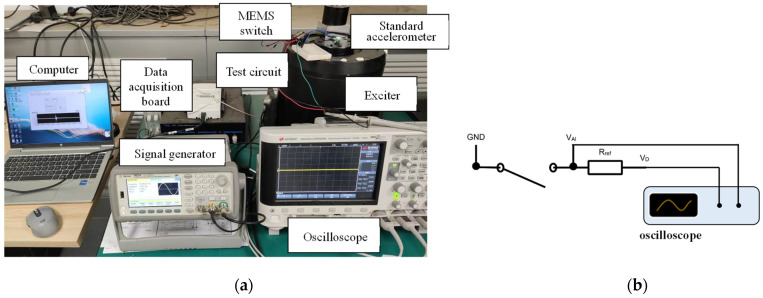
(**a**) The experimental setup of the shaker system; (**b**) test circuit of the electrical performance.

The electrical performance was tested with the apparatus shown in Figure 13. The switch was affixed flatly on the moving element of a BRÜEL & KJÆR Mini-shaker Type 481. The direct current power source and the reference resistor were connected to the switch in series to measure the electrical characteristics, as shown in Figure 13b. The switch is closed when the voltage on the reference resistor is over 2 V, while the voltage output of the power source is 3.1 V. The minimum acceleration when the switch is closed is the acceleration threshold.

**Figure 14:**
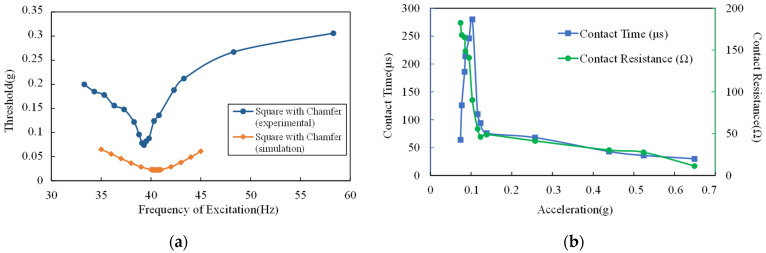
(**a**) Comparison of the simulation result and the experimental result of the acceleration threshold versus the excitation frequency; (**b**) experimental results of the contact time and the contact resistance versus the excitation acceleration.

The experimental results at the excitation frequency from 33 Hz to 59 Hz are shown in Figure 14a. The acceleration threshold is under 39.3 Hz, the excitation is 0.074 g and the threshold is lower than 0.15 g under excitation with 37–41 Hz frequency. The experimental acceleration threshold is higher than the simulation result. However, the dynamic behavior of the proof mass (movable electrode) is basically consistent with the simulation results. It can be speculated that when the acceleration of excitation is small, although the movable electrode can move to the fixed electrode, the contact force between the electrodes is insufficient, resulting in a large contact resistance of the switch, which is not completely closed. Only when the acceleration of the excitation increases to bring in sufficient contact force, the contact resistance of the switch is reduced to below 300 Ω and the voltage across the reference resistor can exceed 2 V, which means the switch is closed.

To verify the speculation above, the contact resistance and ON time of the switch were also measured by the test system in Figure 13a. The frequency of excitation was set as 39.3 Hz. When the switch was stimulated, voltage across the reference resistor was collected to calculate the contact resistance using Equation (17).

(20) $$\frac{V_{ref}}{R_{ref}}=\frac{V-V_{ref}}{R_{con}}$$

Where $V_{ref}$ and $R_{ref}$ are voltage on the reference resistor and its resistance, respectively. $R_{con}$ is the contact resistance and V is the output voltage of the DC power supply (UNI-T, Dongguan, China). As the experimental results show in Figure 14b, the contact resistance decreases as the excitation acceleration increases and the ON time increases at first and then decreases. When the acceleration of the excitation is 0.103 g, the ON time reaches its maximum value, which is 280 μs. When the excitation acceleration exceeds 0.116 g, the contact resistance is less than 50 Ω. Such results show that the contact resistance is closely related to excitation acceleration, which explains the difference between the simulation threshold and the experimental threshold.

**Figure 15:**
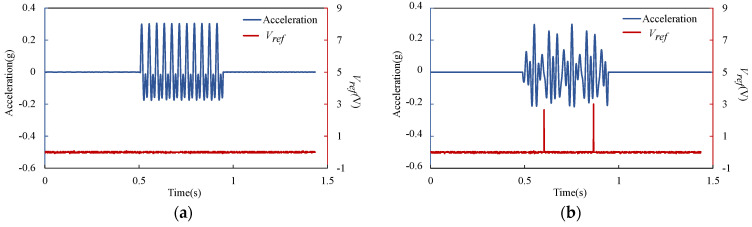
Experimental result of the voltage across the reference resistor and the excitation acceleration in time domain: (**a**) 25 Hz and 50 Hz excitation; (**b**) 25 Hz, 40 Hz and 50 Hz excitation.

The switch was also tested under vibration with multi-frequency. The test system is the same as in Figure 13, the acceleration of the standard accelerometer and the voltage across the reference resistor were collected. Figure 15 shows the test results under excitation with (a) 25 Hz and 50 Hz, and (b) 25 Hz, 40 Hz and 50 Hz. The maximum value of the excitation acceleration in each test remains consistent with a peak value of 0.28 g. The experimental results show that the device has the ability to screen out the vibration component with a specific frequency in the multi-frequency excitation, which is the resonant frequency of the switch. In this experiment, the switch could not be closed under excitation without a 40 Hz vibration component and could be closed under excitation with a 40 Hz vibration component.

**Figure 16:**
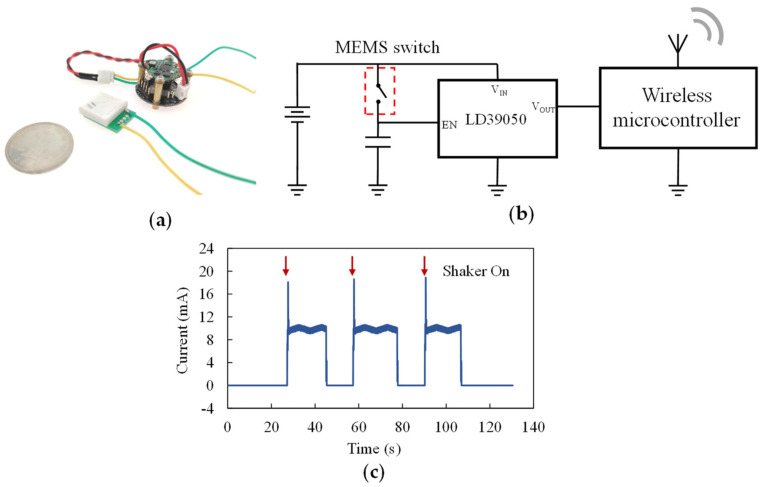
(**a**) A picture of the resonant acceleration switch connected with a sensor node; (**b**) circuit schematic of the sensor node; (**c**) test result of the sensor node’s power supply current.

When the switch is connected to a sensor node, as shown in Figure 16a, the wireless microcontroller of the sensor node will be powered off in its standby state. The circuit schematic of such a sensor node is shown as Figure 16b. To overcome the disadvantage that the switch cannot keep its closingstate, a low dropout regulator (LDO) LD39050 (Stmicroelectronics, Geneva, Switzerland) is used in the sensor node. The capacitor connected with the MEMS switch will be charged as the switch closes when the specific vibration is detected, then the LD39050 will awaken and power the wireless microcontroller. The sensor node was tested using the test system shown in Figure 13. The test results of the sensor node’s power supply current are shown in Figure 16c. The average standby current of the whole sensor node is 4.09 nA, so that the power consumption is only 14.7 nW. A working mode that uses the acceleration switch as a vibration detector reduces the standby power consumption of the sensor nodes by a great extent.

## 5. Discussion

From the results of the experiment, we understand that the proposed switch still has some limitations, such as the contact characteristics. The contact resistance of the switch is larger than 50 Ω when the acceleration is lower than 0.116 g. The contact of the electrodes is unstable as the movable electrode needs to contact the two fixed electrodes at the same time for conduction to occur. In the future, the contact process of the switch will be analyzed and optimized. Further applications of the switch will also be explored, such as the combinations of switches using AND and OR logic to detect complicated vibration signatures.

## 6. Conclusions

In this paper, a resonant acceleration MEMS switch aiming for a low-g acceleration threshold and a low resonant frequency was designed, fabricated and tested. The patterns and geometric dimensions of the switch were designed for a low threshold and a resonant frequency. The stress concentration and eigenfrequencies were also simulated and optimized. The acceleration threshold of the proposed switch was 0.074 g. The resonant frequency turned out to be as low as 39.3 Hz. The standby power consumption of the switch was zero power as it was physically opened in its standby state. The switch was demonstrated to be able to work in a completed sensor node that responded to vibration with specificcharacteristics.

## Figures and Tables

**Table 1:** Structure parameters.

Description	Parameter
Side length of device	*A*
Side length of mass	L
Beam thickness	H
Beam width	W
Interval of beams	d
Young’s modulus of silicon	E
Shear modulus of silicon	G
Moment of inertia of beam	$I_{s}$
Polar moment of inertia of the beam	$I_{p}$

**Table 2:** Geometric parameters.

Description	Value
Side length of device	6 mm
Thickness of mass	50 μm
Beam thickness	50 μm
Beam width	40 μm
Interval of beams	20 μm

## Appendix A

Detailed Simulation

The simulation was conducted using COMSOL Multiphysics. Firstly, gravity was loaded onto the whole device. Then, a sinusoidal prescribed acceleration was added onto the anchor of the cantilever beams for the simulation vibration excitation. A free tetrahedral mesh was used in the model. Finally, we studied the performance of the switch in a stationary, frequency domain and using time dependent steps.
